# Progress on the regulation of colorectal cancer and cardio-oncology by the gut microbiota and nutrients

**DOI:** 10.3389/fimmu.2026.1908629

**Published:** 2026-09-18

**Authors:** Aibo Liu, Rui Tan, Xiaoli Zhou, Peng Wang, Xiao Wang

**Affiliations:** 1 Department of Laboratory Medicine, Sichuan Academy of Medical Sciences and Sichuan Provincial People’s Hospital, Chengdu, China; 2 Genetic diseases Key Laboratory of Sichuan Province, Sichuan Clinical Research Center for Laboratory Medicine, Key Laboratory of Experimental Diagnostic Medicine designated by Health Commission of Sichuan Province, Chengdu, China; 3 Department of Laboratory Medicine, West China School of Medicine, Sichuan University, Sichuan University affiliated Chengdu Second People's Hospital, Chengdu Second People's Hospital, Chengdu, Sichuan, China; 4 Department of Cardiology, The General Hospital of Western Theater Command, Chengdu, Sichuan, China

**Keywords:** cardio-oncology, cardiovascular toxicity, colorectal cancer, gut microbiota, nutrition

## Abstract

As one of the most common malignant tumors worldwide, the pathogenesis of colorectal cancer (CRC) was complex. Recent studies have revealed that dysbiosis of the gut microbiota and abnormal nutritional metabolism played a key role in the occurrence and development of tumors. With the rapid development of cardio-oncology, the cardiovascular toxicity risks faced by CRC patients during radiotherapy, chemotherapy, targeted therapy, and immunotherapy have become increasingly prominent, emerging as a key factor affecting long-term patient outcomes. However, there was currently a lack of systematic integration regarding the mechanisms underlying the interplay between the gut microbiota, nutritional interventions, and cardio-oncology in the management of CRC. This article aims to review the mechanisms underlying the interaction between CRC and the gut microbiota, the effects of nutritional interventions on the gut microbiota and tumor progression, and the important role of cardio-oncology in the treatment of CRC. This review examines the emerging evidence on how gut microbiota and nutrition interact to influence CRC development, progression, and therapeutic response, and proposes a theoretical basis for integrating these insights into multidisciplinary comprehensive treatment strategies.

## 1 Introduction

Colorectal cancer (CRC) is one of the leading causes of cancer incidence and mortality worldwide. Its pathogenesis is highly complex and involves the interaction of multiple factors, including genetic susceptibility, environmental exposures, and lifestyle (1). In recent years, the gut microbiota—as a key “bridge” connecting the environment and the host—has garnered increasing attention for its central role in the development of CRC. The gut microbiota is a complex community of microorganisms residing in the human digestive tract, played an indispensable role in maintaining intestinal homeostasis and regulating the host’s immune and metabolic functions (2). However, when this symbiotic balance is disrupted and dysbiosis occurs, it drives the development and progression of CRC through various mechanisms (3). Specifically, dysbiosis can disrupt the intestinal mucosal barrier, leading to chronic inflammation and the release of carcinogenic metabolites and toxins, thereby creating a microenvironment conducive to tumor development (4). Numerous studies have confirmed that, compared to healthy individuals, CRC patients exhibited significantly reduced diversity and abundance of gut microbiota, as well as marked changes in microbial composition. This was characterized by a decrease in beneficial bacteria (e.g., butyrate-producing bacteria) and an enrichment of harmful bacteria (e.g., enterotoxin-producing *Bacteroides fragilis*) (5, 6). These changes in the microbiome were not only associated with tumor initiation but were also closely linked to tumor progression, metastasis, and treatment response (7, 8). Emerging evidence indicates that the abundance of certain gut microbial communities is predictive of response to neoadjuvant chemoradiotherapy in patients with locally advanced CRC (9, 10). From a therapeutic perspective, adjuvant supplementation with selective probiotics, notably *Rikenella*, along with their derived metabolite propionate, appears to synergistically augment the anti-tumor activity of radioactive hydrogels and immunotherapeutic agents (11). In addition, obesity-related alterations in the gut microbiota have also been suggested to potentially create a microenvironment favorable for tumorigenesis (12).

Nutritional factors played a crucial role in the prevention and treatment of CRC. Epidemiological evidence suggested that unhealthy dietary habits, such as a “Western-style diet” high in fat and red meat and low in fiber, were significant risk factors for CRC (13). Conversely, a diet rich in dietary fiber, whole grains, and fruits and vegetables has a protective effect. The impact of these dietary components on CRC is largely achieved by modulating the composition and function of the gut microbiota (1). For example, a high-fiber diet provided fermentation substrates for the gut microbiota, promoting the growth of bacteria that produce short-chain fatty acids (SCFAs), such as *Bifidobacterium*, *Lactobacillus*, and *Bacteroides*. SCFAs not only provided energy to intestinal epithelial cells, but also exhibited anti-inflammatory, antiproliferative, and tumor cell apoptosis-inducing effects (14, 15). Retrospective studies have shown that in CRC patients who adhere to an anti-inflammatory diet, the abundance of beneficial bacteria in the gut—such as *Bifidobacterium*, *Lactobacillus*, *Prevotella*, and *Akkermansia muciniphila*—significantly increased, while pathogenic bacteria such as *Clostridium nucleatum* and *Clostridium difficile* decreased, accompanied by a significant decrease in inflammatory markers (e.g., CRP and IL-6) and tumor markers (e.g., CEA) (15). In addition, calorie restriction has been shown to inhibit tumor growth in mouse models of CRC by reshaping the gut microbiota and increasing the proportion of *Lactobacillus* (16). Conversely, high-fat diet-induced dysbiosis promoteed lymphangiogenesis and lymph node metastasis in CRC by inhibiting the propionate/GPR41 signaling pathway (17). Therefore, modulating the gut microbiota and restoring microbial balance through nutritional interventions has emerged as a highly promising adjunctive strategy in the prevention and treatment of CRC (1).

Oncocardiology, as an emerging interdisciplinary field, focuses on the short- and long-term adverse effects of anticancer therapies (e.g., chemotherapy, radiation therapy, targeted therapy, and immunotherapy) on the cardiovascular system (18). With the significant extension of survival time for CRC patients, treatment-related cardiovascular toxicity has become a major issue affecting patients’ quality of life and long-term prognosis. The commonly used chemotherapy drugs for CRC patients, such as 5-fluorouracil (5-FU) and oxaliplatin based regimens, as well as targeted drugs such as monoclonal antibodies against vascular endothelial growth factor (VEGF), all have clear risks of cardiac toxicity. 5-FU can cause coronary spasm, myocardial ischemia, and even myocardial infarction (19), while oxaliplatin may cause arrhythmias (20). In addition, pelvic radiotherapy raises long-term risks of radiation-induced heart disease and coronary artery disease via scattered cardiac radiation exposure and subsequent vascular endothelial injury (21). Dysbiosis of the gut microbiota was not only closely associated with the development of CRC, but may also played a role in the development of anticancer therapy-related cardiotoxicity by influencing the host’s metabolic and immune status. For example, systemic inflammation and metabolic disorders (e.g., abnormal cholesterol metabolism) caused by dysbiosis are independent risk factors for cardiovascular disease (22). Therefore, when developing comprehensive treatment plans for CRC, cardiovascular protection strategies must be taken into account, including pre-treatment cardiovascular risk assessment, monitoring of cardiac function during treatment, and long-term follow-up after treatment. At the same time, exploring the possibility of mitigating treatment-related cardiac toxicity by modulating the gut microbiota is a highly promising area of research. This review will provide an in-depth analysis of various aspects, including the molecular mechanisms linking the gut microbiota to CRC, the role of nutritional interventions in modulating the microbiota, and treatment monitoring and protective strategies from the perspective of cardio-oncology. The aim is to establish a multidisciplinary integrated management framework encompassing microbiomics, nutrition, and cardiovascular protection, thereby providing new insights and evidence for the precision prevention and treatment of CRC.

## 2 Methods

A thorough online database search of PubMed/MEDLINE was implemented up to May 2026 to screen qualified primary studies addressing the research topic. The publication time frame was confined to studies published from 1977 to the present. All included records were restricted to articles written in English. Records satisfying the inclusion criteria comprised clinical practice guidelines, clinical trial reports, review papers, and primary clinical research investigating nutritional determinants, gut microbiota profiles, and therapy-related cardiovascular toxicities among individuals with CRC. The PubMed/MEDLINE search combined terms related to colorectal cancer or CRC, gut microbiota or gut microbiome, nutrition, cardio-oncology, and cardiovascular toxicity.

Exclusion criteria: investigations involving subjects suffering from malignant tumors excluding CRC, studies with duplicated publications, studies with incomplete or unclear data regarding relevant outcomes.

### 2.1 Molecular mechanisms underlying the development and progression of CRC

#### 2.1.1 Chronic inflammation driven by dysbiosis and its links to carcinogenesis

An imbalance in the gut microbiota is a key factor in the development of CRC; its primary mechanism involves activating host immune receptors and inducing chronic inflammation, thereby providing a microenvironmental foundation for tumorigenesis. In healthy hosts, the gut microbiota maintains a symbiotic balance with the host; however, once this balance is disrupted—particularly when the ratio of *Firmicutes* to *Bacteroidetes* (F/B) increases—it has been demonstrated to be closely associated with an elevated risk of CRC (23), at the same time, an imbalance in the gut microbiota disrupts the intestinal barrier, ultimately increasing the risk of CRC (23–25) (**Figure 1**).

**Figure 1:**
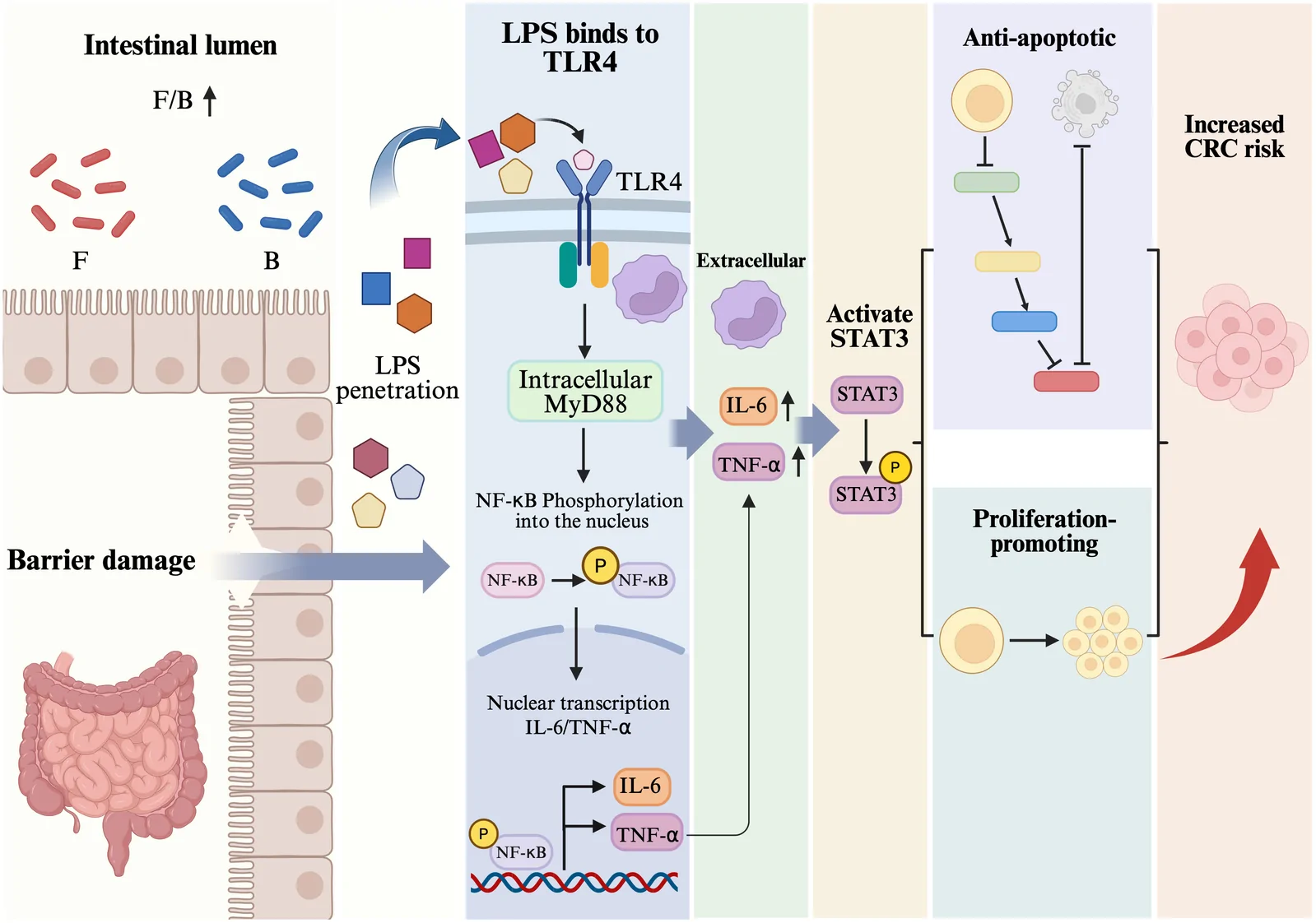
TLR4/MyD88/NF-κB axis mediates gut microbiota dysbiosis-induced CRC. An imbalance in the F/B ratio can disrupt the intestinal barrier function, allowing bacterial components such as LPS to readily penetrate the mucosal layer and activate TLR and NOD-like receptor pathways on the surface of intestinal epithelial cells and immune cells. Activation of TLR4 initiates a downstream MyD88-dependent signaling cascade, leading to the phosphorylation and nuclear translocation of NF-κB, thereby upregulating the expression of pro-inflammatory cytokines such as IL-6 and TNF-α. These cytokines not only promote chronic inflammation of the intestinal mucosa but also activate signaling pathways such as STAT3, inhibiting apoptosis and promoting epithelial cell proliferation, thereby creating a microenvironment conducive to tumorigenesis. Studies have shown that modulating the microbial community structure using *Clostridium butyricum* or a compound of traditional Chinese medicine polysaccharides can reduce the F/B ratio, inhibit the activation of the NF-κB pathway, and consequently alleviate colitis and tumor burden. LPS, lipopolysaccharide; MyD88, myeloid differentiation primary response 88; NOD, nucleotide-binding oligomerization domain; NF-κB, nuclear factor kappa-B; TLR, toll-like receptor; IL, interleukin; TNF-α, tumor necrosis factor; STAT3, signal transducer and activator of transcription 3. Created in https://BioRender.com.

In addition to dysbiosis, the colonization of specific pathogenic bacteria plays a more direct role in the development of CRC. *Fusobacterium nucleatum* and enterotoxigenic *Bacteroides fragilis* (ETBF) are the two most extensively studied carcinogenic bacteria (26, 27). *Bacteroides fragilis* toxin (BFT) cleaves E-cadherin, leading to disruption of the intestinal barrier and thereby promoting cell proliferation and tumorigenesis (28–30). On the other hand, ETBF promoting the progression and metastasis of CRC (31, 32) (**Figure 2**).

**Figure 2:**
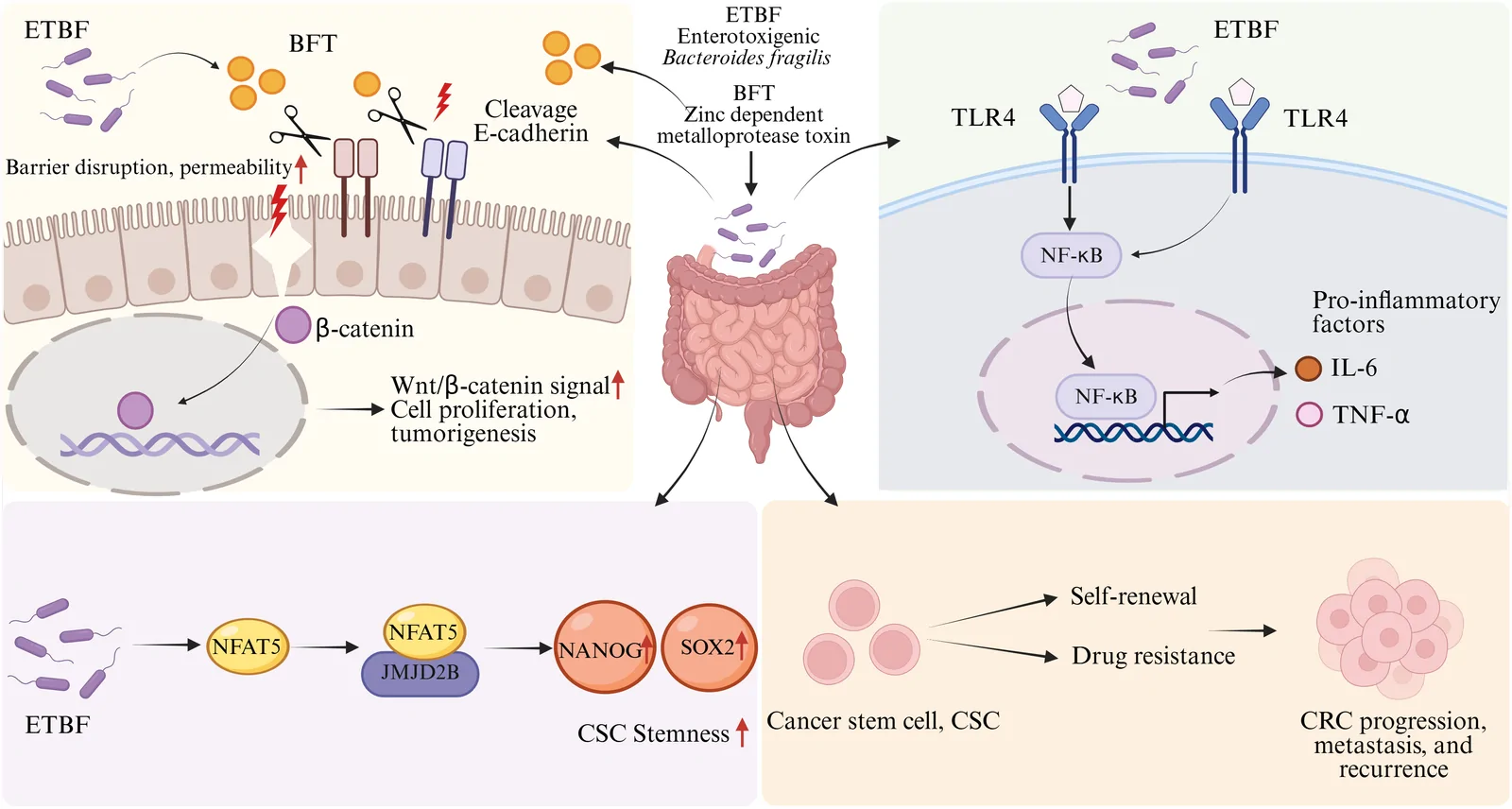
ETBF fuels CRC progression and metastasis via both BFT-dependent and BFT-independent signaling. ETBF secretes its primary virulence factor—BFT, a zinc-dependent metalloproteinase—which is capable of directly cleaving E-cadherin on intestinal epithelial cells. The cleavage of E-cadherin not only disrupts the integrity of the intestinal barrier; but also releases β-catenin into the cell nucleus, activating the Wnt/β-catenin signaling pathway, thereby leading to cell proliferation and tumorigenesis; simultaneously, the cleavage of E-cadherin also activates the NF-κB signaling pathway, exacerbating the inflammatory response. ETBF enhances tumor stem cell properties by upregulating the histone demethylase JMJD2B via the NFAT5 pathway, thereby upregulating transcription factors such as NANOG and SOX2, and promoting the progression and metastasis of CRC. NF-κB, nuclear factor kappa-B; JMJD2B, jumonji domain-containing protein 2B; NFAT5, nuclear factor of activated T-cells 5; NANOG, nanog homeobox; SOX2, SRY-box transcription factor 2. IL, interleukin; TLR, toll-like receptor; TNF-α, tumor necrosis factor. Created in https://BioRender.com.

*Fusobacterium nucleatum* binds to E-cadherin on epithelial cells via its adhesion molecule FadA, thereby activating the β-catenin signaling pathway and upregulating the expression of pro-inflammatory genes (33, 34). Studies have shown that the detection rates and abundance of ETBF and *Fusobacterium nucleatum* in tumor tissues from CRC patients are significantly higher than in healthy controls, and are associated with patient prognosis (35, 36). Metabolites produced by the gut microbiota are key factors in dysbiosis and the development of CRC. In cases of dysbiosis, particularly when influenced by factors such as a high-fat diet, the metabolic patterns of gut microbes undergo changes, leading to increased levels of harmful metabolites such as secondary bile acids (e.g., deoxycholic acid) and hydrogen sulphide (H_2_S). This, in turn, induces the production of reactive oxygen species (ROS), causing oxidative damage to DNA and thereby leading to the malignant transformation of intestinal epithelial cells (26, 37). *Escherichia coli* carrying the pks gene is capable of synthesizing the colibactin genotoxin, which can cause double-strand breaks in DNA and characteristic mutations, such as mutations at specific sites in the APC gene (38, 39). These metabolites and genotoxins act in concert to induce oxidative stress, inhibit DNA repair and directly damage genetic material, thereby playing a significant role in the malignant transformation of rectal epithelial cells. Consequently, the chronic inflammation resulting from dysbiosis, the carcinogenic effects of specific pathogens and the genotoxicity of harmful metabolites collectively constitute the complex relationship between intestinal microecological imbalance and the development of CRC.

The gut microbiota also influences antitumor immune responses by modulating the host immune system, thereby affecting the efficacy of immunotherapy (40, 41). Studies have revealed that microbial quorum sensing plays a crucial role in CRC metastasis, and that targeting microbial communication may represent a novel therapeutic strategy (42). In addition, sex-biased gut microbiota and metabolites also contribute to the sex disparities in CRC incidence (43, 44). Although it remains unclear whether alterations in the gut microbiota are a cause or a consequence of CRC, accumulating evidence suggests that the tumor microenvironment—characterized by localized inflammation and nutrient alterations—can selectively shape the microbial community, thereby establishing a feedback loop that further accelerates tumor progression (13, 45). Therefore, the interplay between the gut microbiota and CRC is dynamic and complex. A thorough understanding of the molecular mechanisms by which gut dysbiosis drives CRC is of great clinical significance for the development of microbiota-based diagnostic biomarkers and intervention strategies (46).

#### 2.1.2 The regulation of the tumor immune microenvironment by microbial metabolites

The gut microbiota interacts with the host immune system through its metabolic products, thereby influencing the composition and function of the TIME. SCFAs, particularly butyrate—the primary product of dietary fiber fermentation by the gut microbiota—play a significant role in the immune regulation of CRC. Firstly, butyric acid suppresses anti-tumor immunity (47, 48); Secondly, high concentrations of butyric acid exert an anti-tumor effect (49) (**Figure 3**).

**Figure 3:**
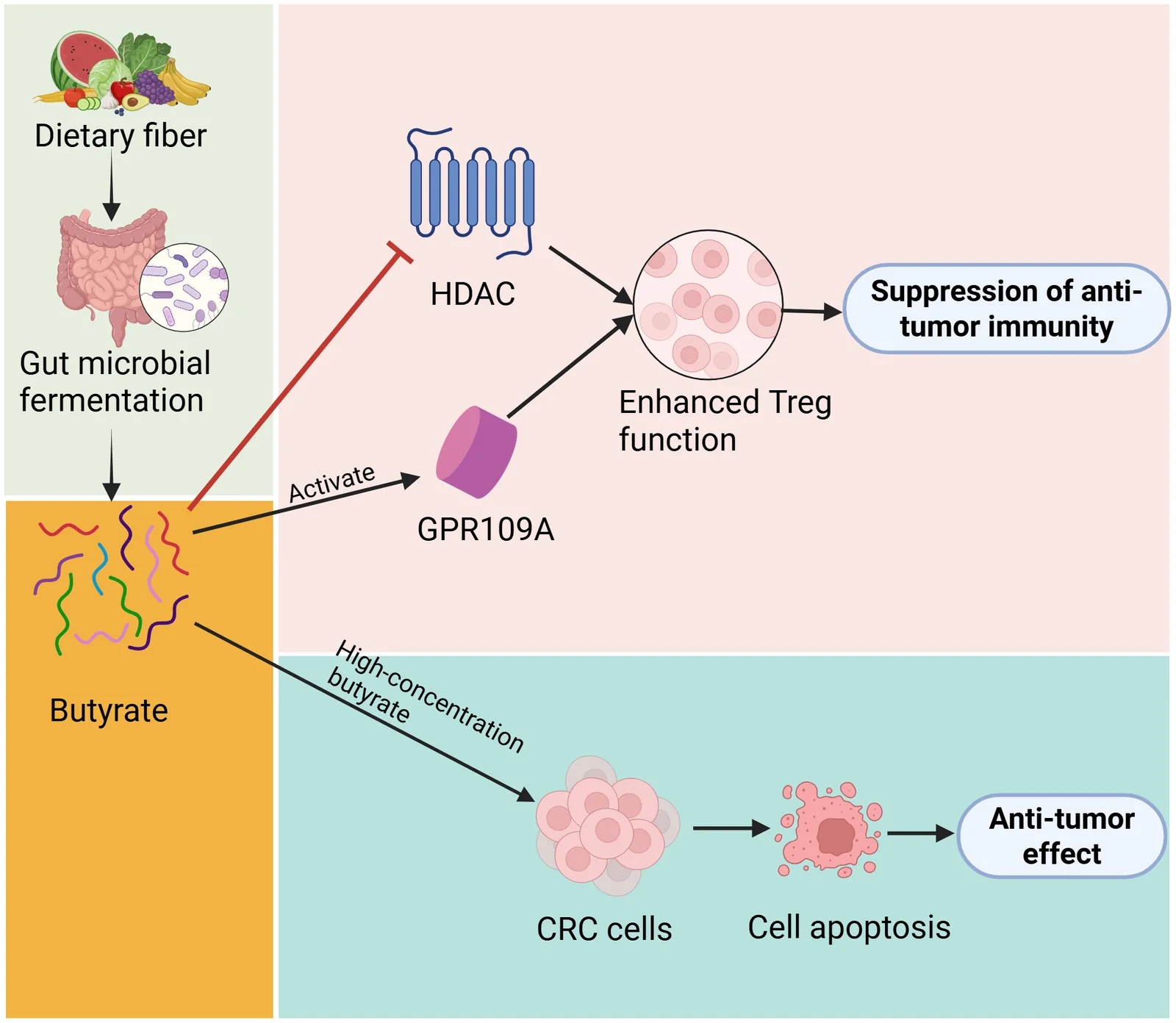
Dual immunomodulatory effects of butyrate in CRC. Butyrate produced by gut microbial fermentation of dietary fiber, exerts dual and opposing effects on CRC immunity in a concentration-dependent manner. At relatively low concentrations, butyrate enhances the suppressive function of Tregs through two convergent mechanisms: (i) inhibition of HDACs, and (ii) activation of GPR109A. This Treg-focused immunomodulation dampens antitumor immune surveillance, thereby creating an immunosuppressive tumor microenvironment that may favor immune evasion. In contrast, at high concentrations, butyrate directly induces CRC cell apoptosis, exerting intrinsic antitumor effects. Collectively, this concentration-dependent duality positions SCFAs, particularly butyrate, as a critical mechanistic link bridging dietary patterns, gut microbiota composition, and host antitumor immunity. Tregs, regulatory T cells; HDACs, histone deacetylases; GPR109A, G protein-coupled receptor 109A; SCFAs, short-chain fatty acids. Created in https://BioRender.com.

Research has found that the levels of butyrate and acetate in the feces of CRC patients are significantly lower than those in healthy individuals, while the difference in propionate levels is not significant, suggesting that SCFA-producing gut microbiota are closely associated with the development and progression of CRC (50, 51). It has been demonstrated that increasing the production of SCFAs through the supplementation of probiotics or dietary fiber (e.g., pectin and inulin) enhances the efficacy of anti-PD-1 immunotherapy; this mechanism is associated with the promotion of CD8+ T cell infiltration and activation within the tumor microenvironment (52, 53). In addition to SCFAs, tryptophan is metabolized by the gut microbiota to produce compounds such as indole-3-carbaldehyde (IAld), which activate aryl hydrocarbon receptor (AhR), thus preserving intestinal barrier integrity while orchestrating antitumor immune responses (54) (**Figure 4**).

**Figure 4:**
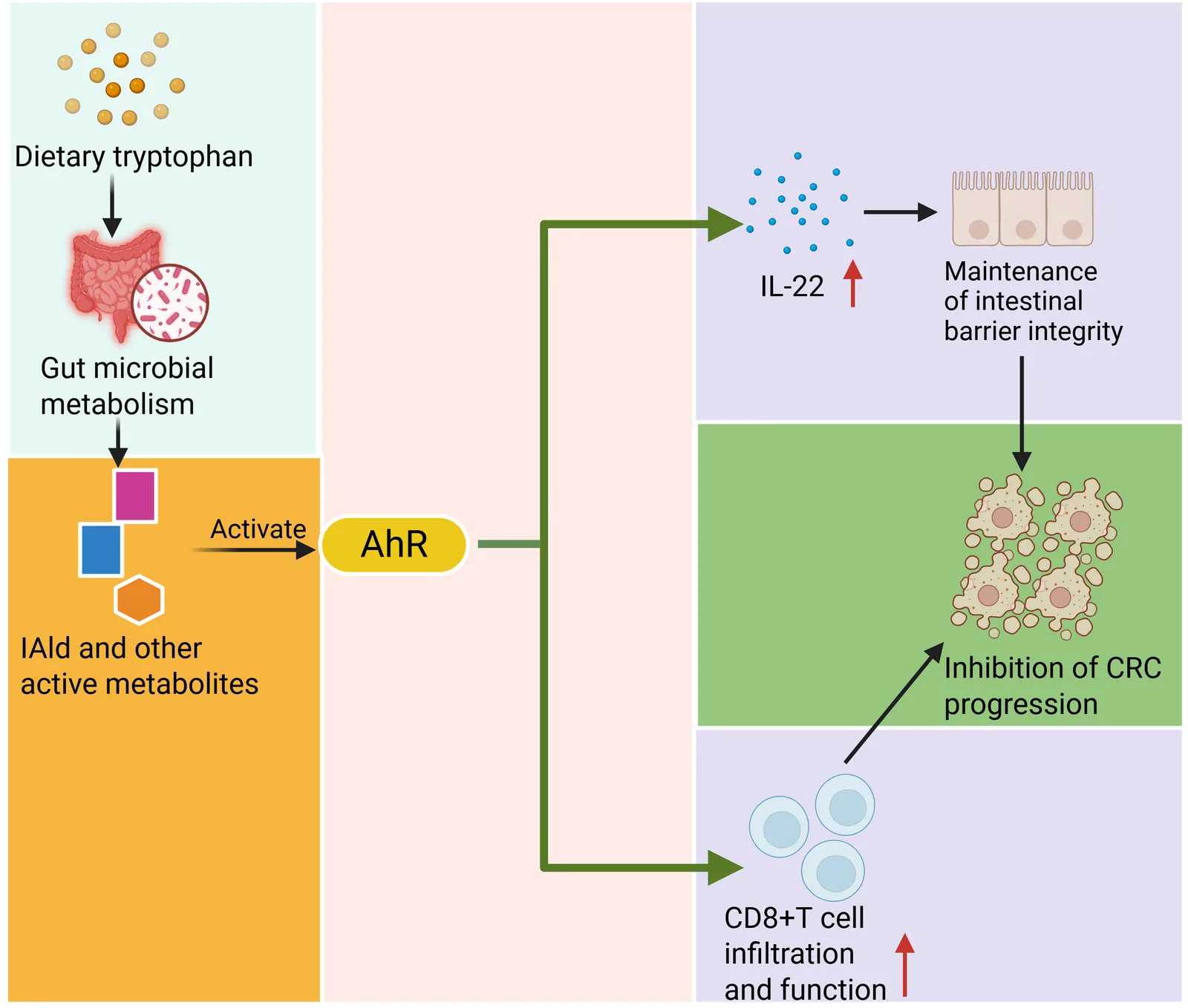
Gut microbial tryptophan metabolism restricts CRC by AhR-mediated dual protection of intestinal barrier and antitumor immunity. Tryptophan metabolites are also key molecules through which the microbiota regulates the TIME. The gut microbiota metabolizes tryptophan from food into various bioactive compounds, such as IAld. These bioactive compounds promote the secretion of IL-22 by activating the AhR, thereby maintaining the integrity of the intestinal barrier, which is crucial for limiting tumor progression. Furthermore, activation of the AhR signaling pathway can influence the infiltration and function of CD8+ T cells, thereby modulating the anti-tumor immune response. AhR, aryl hydrocarbon receptor; IAld, indole-3-carbaldehyde; IL, interleukin; CD8+ T cells, CD8-positive T lymphocytes. Created in https://BioRender.com.

However, microbial metabolites are not always beneficial; for example, LPS derived from the microbiota is a potent pro-inflammatory factor which, by activating myeloid-derived suppressor cells (MDSCs), impairs the anti-tumor activity of natural killer (NK) cells and cytotoxic T lymphocytes, thereby promoting tumor immune evasion (55, 56). The formation of this immunosuppressive microenvironment is one of the factors contributing to the progression of CRC and the development of resistance to immunotherapy. Consequently, the regulation of the TIME by gut microbiota metabolites is a complex process, the effects of which depend on the concentration of the metabolites, the local microenvironment, and the host’s immune status. Understanding the mechanisms of action between these metabolites and immune cells will provide an important theoretical foundation for immunotherapy for CRC based on the regulation of gut microbiota metabolites (57, 58).

### 2.2 Characteristics of the gut microbiome in patients with CRC and their diagnostic applications

#### 2.2.1 Identification of microbiome biomarkers associated with CRC

Based on metagenomic sequencing studies, the gut microbiota structure of CRC patients undergoes significant changes, with a marked increase in the abundance of pathogenic bacteria such as *Fusobacterium nucleatum* and *Peptostreptococcus anaerobius*, while the abundance of beneficial butyrate-producing bacteria, such as *Faecalibacterium prausnitzii* and *Ruminococcus*, is significantly reduced. These synergistic changes in microbial composition provide a crucial basis for the development of non-invasive early screening based on fecal samples. *Fusobacterium nucleatum* and *Peptostreptococcus anaerobius* are significantly associated with the staging and metastasis of CRC, and their combined detection offers the highest diagnostic value (59). Furthermore, research has found that *Parvimonas micra* is significantly enriched in patients with CRC and is closely associated with the development and progression of the disease (60). These microbial biomarkers are not only capable of distinguishing between healthy individuals and patients with CRC, but can also identify precancerous lesions such as rectal adenomas (61). The combination of gut microbiota biomarkers with fecal occult blood tests or circulating tumor DNA testing can significantly improve the sensitivity and specificity of CRC diagnosis; it is particularly valuable for the detection of early-stage lesions. Studies have found that fecal bacterial biomarkers show considerable potential as screening markers in cost-effectiveness analyses (62). Multi-queue metagenomic analysis indicates that the combined use of bacterial and fungal biomarkers can further improve diagnostic accuracy (63). Consequently, the combined use of bacterial and fungal biomarkers offers a more comprehensive approach to the precise screening for CRC.

Research has found significant differences in gut microbiota profiles among CRC patients with different tumor stages and molecular subtypes, suggesting that gut microbiota profiles can be used for prognostic stratification in CRC; for example, patients with BRAF^V600E^-mutated CRC exhibit a unique fecal microbiota profile, with microbiota characteristics more closely resembling those of healthy individuals than those of BRAF wild-type patients; *Prevotella enoeca* and *Ruthenibacterium lactatiformans* are the most potent discriminatory markers in BRAF^V600E^ mutant and wild-type patients, respectively (64). Furthermore, the gut microbiota is closely associated with tumor staging. Studies have shown that patients with stage IV CRC exhibit significantly reduced α-diversity in their fecal microbiota, as well as marked alterations in β-diversity. Eleven biomarkers identified through a random forest model—including the significant enrichment of *Peptostreptococcus* and *Parvimonas micra*, which are associated with the progression of CRC, and the significant suppression of *Fenollaria* and *Staphylococcus*, which are associated with remission, and a significant correlation with blood biochemical markers such as carcinoembryonic antigen (CEA) and carbohydrate antigen 19-9 (CA19-9). This suggests that these gut microbiota biomarkers play a synergistic role in the onset and progression of the disease (65). The study also found differences in the composition of the gut microbiota between left-sided and right-sided CRC: *Fusobacterium* is predominant in left-sided CRC, whereas right-sided CRC is dominated by genera such as *Blautia* and *Faecalibacterium* (66). This not only reflects differences in the tumor microenvironment but may also be associated with the biological behavior and prognosis of tumors in different locations. A risk score based on the preoperative gut microbiota can accurately predict disease progression and survival outcomes in patients with CRC, with predictive power that even surpasses that of traditional clinical indicators such as CEA and lymph node infiltration (67). Therefore, in-depth research into stage-specific microbial biomarkers holds significant clinical value for the prognostic assessment and personalized treatment of CRC.

#### 2.2.2 The gut microbiota as a predictor of treatment response

The gut microbiota shows great potential in predicting the response of CRC patients to various treatments; in the field of immune checkpoint inhibitor (ICI) therapy, the composition of patients’ gut microbiota has been shown to be closely associated with treatment efficacy. Research indicates that a gut microbiome environment rich in *Akkermansia muciniphila* and *Bifidobacterium* can enhance the immune system’s anti-tumor activity, leading to a better response in patients to immunotherapies such as programmed death receptor-1 (PD-1) inhibitors (68, 69). However, a gut microbiota dominated by *Bacteroides*, particularly ETBF, is frequently associated with treatment resistance; this may be linked to the chronic inflammation and immunosuppressive microenvironment it induces (2, 70). This correlation between gut microbiota characteristics and treatment efficacy provides a theoretical basis for identifying potential beneficiaries through gut microbiota analysis prior to treatment, thereby facilitating the precision of immunotherapy.

Chemotherapy is the cornerstone of CRC treatment, and the gut microbiota can influence the efficacy of chemotherapeutic agents through the activity of metabolic enzymes. For example, the severe diarrhoea caused by irinotecan is linked to its metabolism by bacterial β-glucuronidase in the gut into the toxic SN-38G. A study of patients with metastatic CRC undergoing irinotecan chemotherapy demonstrated that probiotic intervention can reduce intestinal β-glucuronidase activity, thereby significantly reducing the incidence of diarrhoea in specific patient groups (e.g., those with stomas) (71). Certain bacterial strains, such as *Escherichia coli* carrying the polyketase synthase (pks) gene island, are capable of producing the genotoxin colibactin, which induces double-strand breaks in DNA. This may not only promote tumorigenesis but also interfere with DNA-targeting chemotherapeutic agents (e.g., 5-FU and oxaliplatin) (72, 73). Consequently, assessing the activity of specific metabolites or the abundance of pathogenic bacteria in a patient’s gut microbiota holds promise as a novel biomarker for predicting the efficacy of chemotherapy and its adverse effects.

Radiotherapy is widely used in the treatment of CRC and can cause radiation enteritis. Studies have shown that radiotherapy significantly alters the structure of the gut microbiota, leading to dysbiosis, and that this dysbiosis is associated with the severity of radiation enteritis (74). Changes in the gut microbiota following radiotherapy include a reduction in beneficial bacteria such as *Lactobacillus*, which have a protective effect, and an increase in opportunistic pathogens such as *Enterobacteriaceae* (69, 75), this imbalance in the gut microbiota can disrupt the intestinal mucosal barrier, exacerbate local inflammatory responses, and worsen radiation-induced damage. By analyzing the characteristics of the gut microbiota in patients prior to or during the early stages of radiotherapy, particularly changes in the ratio of *Lactobacillus* to *Enterobacteriaceae*, it may be possible to identify high-risk patients for severe radiation enteritis at an early stage. This would allow for the timely implementation of preventive measures, such as the use of specific probiotics or dietary adjustments, to maintain gut microbiota homeostasis and mitigate adverse effects (70, 76), as a key link between treatment and the host response, the value of the gut microbiota as a predictor of treatment response is becoming increasingly evident, offering new insights for improving the treatment of CRC patients.

### 2.3 Modulatory effects of nutritional factors on gut microbiota and cardiovascular toxicity in CRC patients

#### 2.3.1 The tumor-protective mechanisms of dietary fiber and SCFAs

A high dietary fiber intake is a key strategy for preventing CRC. Studies have found that consuming at least 25 grams of dietary fiber daily—particularly when fermented by the gut microbiota—can significantly increase the production of SCFAs, such as butyrate, in the gut (77). These SCFAs, particularly butyrate, are capable of inhibiting the proliferation of CRC cells and inducing their apoptosis (2, 78), primarily by regulating gene expression through mechanisms such as the inhibition of HDACs (79). At the same time, butyrate, as the primary energy source for colonic epithelial cells, enhances the intestinal barrier function, effectively reducing the entry of harmful substances such as endotoxins into the bloodstream and lowering inflammation levels (80, 81). Research has shown that concentrations of SCFAs in the feces of CRC patients are significantly lower than those in healthy control groups, and this reduction is associated with an increased risk of cancer (50). Therefore, maintaining high levels of SCFAs in the gut through an adequate intake of dietary fiber is key to creating a gut microenvironment that is unfavorable to tumor growth.

Among dietary fibers, soluble fibers (e.g., inulin) exhibit better anti-tumor properties than insoluble fibers. Soluble fibers are more readily fermented by beneficial gut bacteria, thereby promoting the growth of butyrate-producing bacteria such as *Bacteroides* (48, 51). Inulin, a soluble fiber, has been shown to significantly increase the abundance of *Lactobacillus* in the colon of animal models of CRC, raising levels of acetate, propionate and butyrate, thereby inhibiting the development of colon tumors (53). The gut microbiota can convert primary bile acids into secondary bile acids; the latter, such as deoxycholic acid, have been shown to be associated with an increased risk of CRC. Not only do the fermentation products of soluble fiber exert a direct anti-tumor effect, but soluble fiber can also reduce the production of secondary bile acids and their carcinogenic irritation of the rectal mucosa by promoting the growth of beneficial bacteria (82, 83). However, recent studies have suggested that high doses of soluble fiber (e.g., inulin) may promote the development of colorectal tumors by inducing gut microbiota dysbiosis and altering metabolites, highlighting the ‘dose-response relationship’ of fiber intake and the complexity of the gut microbiota (84). Nevertheless, the prevailing view remains that increasing soluble fiber intake is the best way to raise levels of protective SCFAs.

Insufficient dietary fiber intake can lead to a reduction in gut microbiota diversity and disrupt the balance of the gut microbiome, resulting in a decrease in the abundance of beneficial bacteria that produce SCFAs (e.g., *Bifidobacterium*) and an overgrowth of pathogenic bacteria (4, 85). Bacteria closely associated with the development of CRC, such as pks^+^ *E. coli* producing the colibactin gene toxin, are more likely to colonize and proliferate in an environment of dysbiosis (86), these pathogenic bacteria promote tumor development by inducing chronic inflammation, causing DNA damage and suppressing anti-tumor immunity (70, 87). Insufficient dietary fiber intake leads to dysbiosis and a reduction in butyrate production, which weakens the intestinal barrier and increases the permeability of the intestinal mucosa, allowing carcinogens to come into easier contact with epithelial cells and thereby increasing the risk of CRC recurrence (2, 88). Research has found that patients with CRC typically have lower gut microbiota diversity than healthy individuals, and that the abundance of certain pathogenic bacteria is increased (89, 90). Maintaining an adequate intake of dietary fiber to nourish beneficial gut bacteria and suppress pathogenic bacteria is therefore of great importance in preventing CRC and its recurrence.

#### 2.3.2 Cancer-promoting gut microbiota metabolic pathways associated with red and processed meat

Excessive consumption of red and processed meat is considered a major risk factor for CRC, a mechanism closely linked to the metabolic activity of the gut microbiota. Haem iron in red meat and nitrites used as preservatives in processed meat can, under the influence of certain gut microbiota, be converted into highly mutagenic N-nitroso compounds (NOCs). These compounds can bind directly to the DNA of colonic epithelial cells, inducing DNA damage and genetic mutations, thereby initiating the carcinogenic process (91, 92). A diet high in red meat increases the levels of *Desulfovibrio*, which ferments sulfur-containing amino acids to produce H_2_S, contributing to the development and progression of CRC (93–98) (**Figure 5**).

**Figure 5:**
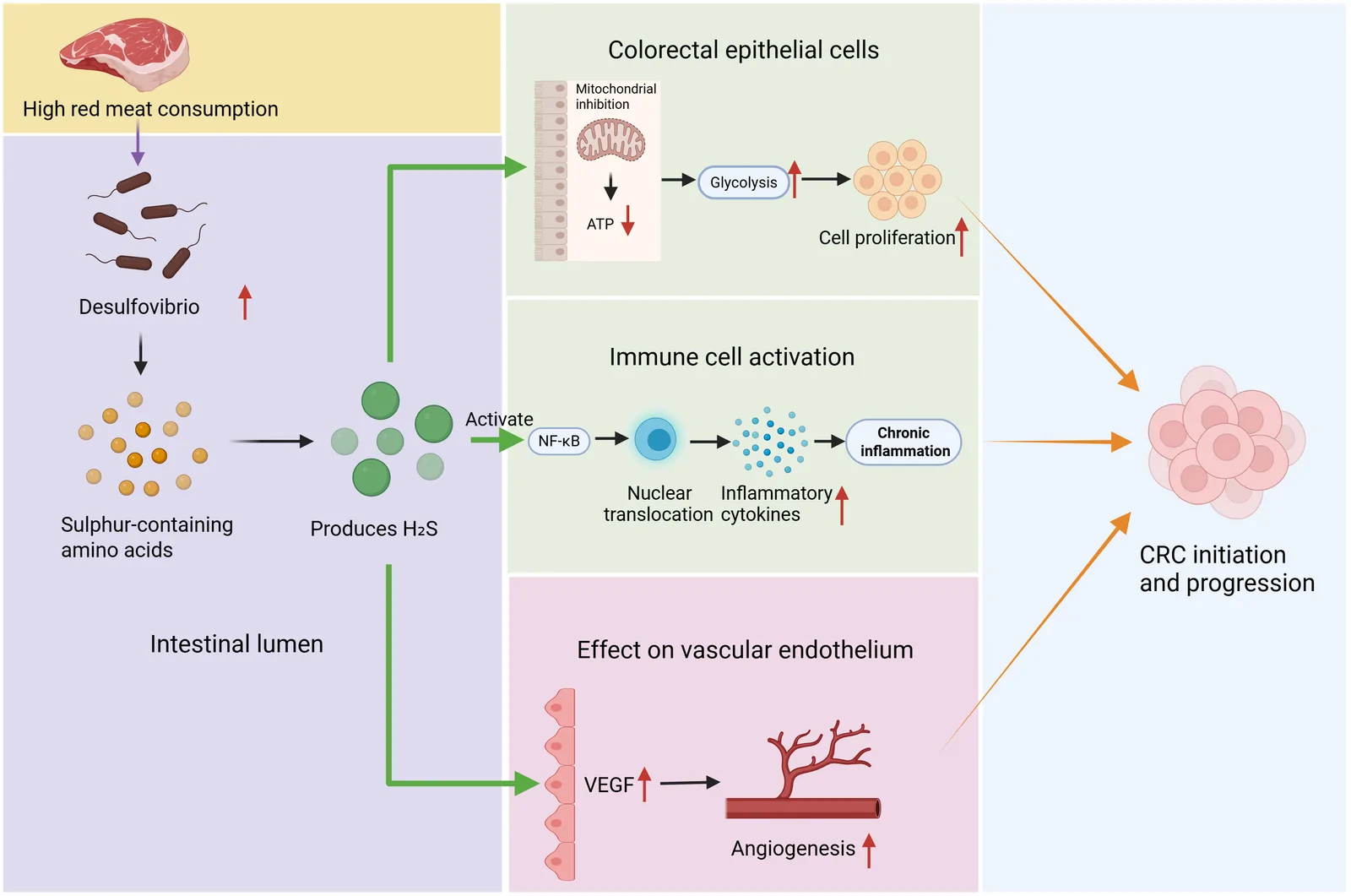
Mechanisms of red-meat diet-driven CRC are mediated by *Desulfovibrio*-derived H_2_S through three distinct pathways. A diet high in red meat significantly alters the composition of the gut microbiota, promoting an increase in the abundance of *Desulfovibrio*, a sulfate-reducing bacterium. The fermentation of sulfur-containing amino acids in the gut by *Desulfovibrio* produces H_2_S. High levels of H_2_S can inhibit the Complex IV in colonic epithelial cells, thereby blocking the oxidative metabolism of SCFAs. This leads to cellular metabolic disruption, forcing colonic cells to switch to glycolysis for energy supply and inducing their excessive proliferation, thereby creating a cellular environment conducive to tumor development. Furthermore, by activating inflammatory signaling pathways such as NF-κB, it induces chronic intestinal inflammation, which is a driving factor in CRC; H_2_S also promotes tumor angiogenesis, providing nutritional support for tumor growth. Consequently, red meat, by inducing the enrichment of sulfate-reducing bacteria, constitutes a cancer-promoting pathway that extends from gut microbiota dysbiosis to excessive H_2_S production, and ultimately to inflammation-driven carcinogenesis. H_2_S, hydrogen sulphide; SCFAs, short-chain fatty acids; NF-κB, nuclear factor kappa-B. ATP, adenosine triphosphate; VEGF, vascular endothelial growth factor; Complex IV, mitochondrial cytochrome c oxidase. Created in https://BioRender.com.

The role of ultra-processed foods (UPF) in modulating the gut microbiota and promoting CRC has gained increasing attention. Resistant starch nanoparticles from certain UPF (e.g., rice-derived RS nanoparticles) can enter intestinal epithelial cells, activate TRPML1 channels, and induce lysosomal enzyme release into the lumen. They also reduce goblet cell numbers, downregulate tight junction proteins, and disturb the gut microbiota, collectively compromising intestinal barrier integrity and promoting colitis (99). Although colitis itself is not directly equivalent to CRC, chronic intestinal inflammation is a well-established risk factor for colorectal carcinogenesis. Therefore, resistant starch nanoparticles derived from UPF may act as a concealed driving factor in CRC development by inducing gut dysbiosis, barrier disruption, and inflammatory responses (99, 100). In studies of patients with stage I–III CRC, higher post-diagnostic intake of UPF has been significantly associated with increased cardiovascular mortality. This association suggests that UPF may influence long-term survival primarily through effects on cardiovascular health rather than by directly promoting cancer progression. Analyses restricted to participants with a history of cancer have further demonstrated a link between UPF consumption and mortality, reinforcing the potential adverse impact of UPF on the cardiovascular system. Collectively, these findings indicate that even among cancer patients, reducing UPF intake may be of considerable importance for lowering cardiovascular risk and improving survival outcomes (101).

A diet based primarily on plant-based foods has demonstrated significant potential for preventing CRC, plant-based diets are rich in polyphenols, flavonoids and antioxidants; these compounds can inhibit the adhesion and colonization of pathogenic bacteria through various mechanisms. For example, polyphenols can disrupt the cell membranes of pathogenic bacteria such as *Fusobacterium nucleatum* or interfere with their binding to host cells, thereby inhibiting their colonization of the intestinal mucosa (102–105). Probiotics (e.g., *Lactobacillus* and *Bifidobacterium*) produce SCFAs through the fermentation of plant-based dietary fiber, in particular butyrate, which is not only the primary energy source for colonic epithelial cells but also exerts anti-inflammatory and anti-tumor effects by inhibiting HDACs and activating G protein-coupled receptors (e.g., GPR109A) (106), A plant-based diet, by promoting the growth of probiotics, can effectively reverse the gut dysbiosis caused by high red meat intake, reduce the proportion of harmful bacteria such as *Desulfovibrio*, restore the balance of the gut microbiota, and attenuate red meat-associated carcinogenic metabolic pathways (107). Therefore, adjusting dietary patterns and increasing the intake of plant-based foods is an effective strategy for the prevention of CRC.

#### 2.3.3 The role of the gut microbiota in the absorption of specific nutrients (e.g., vitamin D and omega-3 fatty acids)

The preventive role of vitamin D in CRC is attracting increasing attention; its mechanism involves not only anti-tumor effects but also plays a significant role through the regulation of the gut microbiota and the immune system (108), Vitamin D can influence the composition of the gut microbiota by regulating the secretion of antimicrobial peptides, such as β-defensins (109). Research indicates that vitamin D deficiency in patients with CRC is closely associated with reduced gut microbiota diversity and a decrease in butyrate-producing bacteria (e.g., *Faecalibacterium prausnitzii*). Daily supplementation with 2000 IU of vitamin D can significantly alter the abundance of various gut microbiota and modulate multiple metabolic pathways; these changes in the microbiota partially mediate the effect of vitamin D in raising serum 25-Hydroxyvitamin D (25(OH)D) levels (110). Animal studies have demonstrated that vitamin D can suppress the excessive activation of the JAK-STAT signaling pathway by activating the vitamin D receptor (VDR), thereby improving gut dysbiosis, restoring butyrate metabolism and rebalancing macrophage polarization, thus inhibiting the development of CRC (111). Epidemiological evidence also supports a negative correlation between serum 25(OH)D levels and the risk of CRC, with this association being particularly pronounced in cases of colon cancer (112, 113). However, some studies have also indicated that no clear causal relationship has been found between circulating vitamin D levels and the risk of CRC, suggesting that the associations observed in observational studies may be influenced by confounding factors (114). Nevertheless, the role of vitamin D in regulating the gut microbiota and its potential in the prevention of CRC warrant further investigation.

Omega-3 polyunsaturated fatty acids (PUFAs), particularly eicosapentaenoic acid (EPA) and docosahexaenoic acid (DHA), have demonstrated multifaceted potential in the prevention and treatment of CRC. Increasing the intake of omega-3 PUFAs can significantly boost the density of butyrate-producing bacteria, thereby enhancing the production of SCFAs in the gut, which possess anti-inflammatory and anti-tumor properties (115). Omega-3 PUFAs can also increase the abundance of *Akkermansia muciniphila* and *Lactobacillus*, reduce the production of LPS, and inhibit the progression of CRC through anti-inflammatory effects and by modulating the immune microenvironment (70, 116, 117). A meta-analysis has shown that circulating EPA levels are significantly inversely associated with the risk of CRC, and that increased EPA levels can reduce this risk (118). However, the anti-cancer effects of ω-3 PUFAs may be modulated by host factors; the expression of intestinal 15-lipoxygenase-1 (ALOX15) is a key host factor determining the direction of the effects of EPA and DHA on CRC. ALOX15 promotes the oxidative metabolism of EPA and DHA to produce prostaglandin E2, thereby inhibiting pro-tumor inflammatory factors (119). Furthermore, DHA can suppress the viability of organoids derived from CRC patients by inducing ferroptosis, thereby offering a new strategy for enhancing the efficacy of chemotherapy (120). Although some studies have shown a positive association between omega-3 fatty acids and the risk of CRC, this may be due to differences in study design and study populations, and further validation is required (121).

As an important trace element, selenium also plays a significant role in the prevention of CRC. By enhancing the activity of glutathione peroxidase (GPx), selenium protects intestinal epithelial cells from oxidative damage while promoting the growth of butyrate-producing bacteria; its anti-tumor effects are partly dependent on metabolic regulation mediated by the gut microbiota (122–125). Research has found that a higher dietary selenium intake prior to CRC diagnosis is associated with a reduced risk of postoperative mortality in CRC patients, suggesting that selenium may serve as an adjunctive dietary factor in improving survival outcomes for CRC patients (126). The anti-cancer mechanisms of selenium involve multiple pathways, including the regulation of the cell cycle, the induction of apoptosis, and the enhancement of immune surveillance. Furthermore, selenium can influence the composition of the gut microbiota, and certain gut bacteria are capable of metabolizing selenium to produce bioactive selenium metabolites; these compounds can inhibit tumor development by modulating the intestinal microenvironment (127–129). Although there is evidence to support the potential value of selenium in the prevention of CRC, the results of randomized controlled trials on selenium supplementation have been inconsistent, with some studies failing to identify a significant protective effect (130). Therefore, the recommended intake of selenium and the mechanisms underlying its synergistic interaction with the gut microbiota still need to be elucidated through larger-scale clinical studies.

To enhance readability and facilitate future research, we have summarized the clinical studies on the role of vitamin D, omega-3 PUFAs and selenium in the prevention of CRC (**Table 1**).

**Table 1:** The history of vitamin D, omega-3 PUFAs and selenium in the prevention of CRC.

Time	Sex(M/F)	Country	Main biomarkers	Total population	Representative target	Subject	Main treatments	Study design	Main outcome
1980	M	USA	N/A	N/A	Vitamin D	Human	N/A	Ecological study/Correlational study	Put forward Hypothesis: Vitamin D is protective against CRC (131)
1985	M	USA	N/A	1954	Vitamin D	Human	N/A	Prospective cohort study	Dietary vitamin D intake is inversely associated with CRC risk in prospective cohort studies (132)
1989	M/F	USA	25(OH)D	101	Vitamin D	Human	N/A	Prospective nested case-control study	Serum 25(OH)D is inversely associated with CRC risk (133)
1990	M/F	Finland	selenium	3,073	Selenium	Human	N/A	Prospective nested case-control study	Serum selenium is inversely associated with gastric and lung cancer risk (134)
1991	M	USA	N/A	273	ω-3 PUFAs	Animal	High fish oil (ω-3) vs. high corn oil (ω-6)	Animal experiment/Interventional study	High ω-3/ω-6 ratio diet inhibits chemically induced colon tumors in rats (135)
1996	M/F	USA	selenium	1,312	Selenium	Human	Oral selenium	Randomized, double-blind, placebo-controlled trial	Selenium supplementation reduces the incidence of CRC (136)
1997	M	Finland	25(OH)D/1,25(OH)_2_D	436	Vitamin D	Human	N/A	Prospective nested case-control study	Serum 25(OH)D is inversely associated with CRC risk (137)
2006	M	USA	25(OH)D	47,800	Vitamin D	Human	N/A	Prospective cohort study	Higher predicted vitamin D status is associated with reduced risk of digestive-system cancers, including CRC (138)
2016	M/F	USA	N/A	125,172	ω-3 PUFAs	Human	N/A	Prospective cohort study	Marine ω-3 PUFAs intake is inversely associated with FOXP3^+^ T-cell-high CRC risk (139)
2017	M/F	USA	N/A	1,659	ω-3 PUFAs	Human	N/A	Prospective cohort study	Post-diagnostic marine ω-3 PUFAs intake is associated with reduced CRC-specific mortality (140)
2017	N/A	USA	N/A	80	Selenium	Animal	Dietary selenium supplementation	Animal experiment/Interventional study	Selenium protects against inflammation-associated CRC in mice (123)
2021	M/F	China	25(OH)D	360,061	Vitamin D	Human	N/A	Prospective cohort study	Serum 25(OH)D is linearly and inversely associated with CRC risk (113)
2022	M/F	Italy	25(OH)D	60	Vitamin D	Human	Vitamin D_3_ 2000 IU/day	Phase II randomized, double-blind, placebo-controlled trial	Vitamin D supplementation alters gut microbiota, which mediates the effect on 25(OH)D levels in CRC survivors (110)
2023	M/F	Poland	N/A	671	Selenium	Human	N/A	Prospective observational cohort study	Higher pre-diagnostic dietary selenium intake is associated with improved overall survival in CRC patients, particularly in lymph node-positive cases (126)
2023	M/F	China	25(OH)D	403,170	Vitamin D	Human	N/A	Prospective cohort study	Serum 25(OH)D is inversely associated with CRC risk in a dose-dependent manner (112)
2024	M	China	N/A	25	Selenium	Animal	FMT; sodium selenite (3 mg/kg, i.p.); FMT + sodium selenite	Animal experiment/Interventional study	Sodium selenite inhibits CRC development in mice (125)
2024	M	China	N/A	45	Selenium	Animal	Mannose; C/S-Se (SeNPs in CS/SA hydrogel); C/S-MSe (mannose-functionalized SeNPs in CS/SA hydrogel)	Animal experiment/Interventional study	Selenium nanoparticles alleviate colitis via NF-κB suppression and GPX upregulation, indicating a protective role against inflammation-driven CRC (124)
2025	M/F	China	LC ω-3 PUFAs	1,663,721	ω-3 PUFAs	Human	N/A	Systematic review and dose-response meta-analysis	Circulating EPA is inversely associated with CRC risk in a linear dose-dependent manner (118)
2026	M/F	USA	N/A	60	Vitamin D	Human	1,25(OH)_2_D_3_ (*in vitro*)	Ex vivo functional genomics study	Ancestry-related genetic variants influence vitamin D response, revealing new targets for personalized prevention (141)
2026	M/F	China	N/A	2,889	ω-3 PUFAs	Human	ω-3 PUFAs supplementation	Systematic review and meta-analysis	Perioperative ω-3 PUFAs supplementation reduces inflammation, enhances immune function, and shortens hospital stay (142)

N/A, Not applicable; M, male; F, female; PUFAs, polyunsaturated fatty acids; 25(OH)D, 25-Hydroxyvitamin D; ω, Omega; 1, 25(OH)_2_D, 1, 25-Dihydroxyvitamin D; FOXP3, forkhead box P3; NF-κB, nuclear factor kappa-B; GPX, glutathione peroxidase; FMT, fecal microbiota transplantation; EPA, eicosapentaenoic acid; LC, long-chain; Se, selenium; NPs, nanoparticles; CS, chitosan; SA, sodium alginate; C/S-MSe, colon-targeted hydrogel embedded M-SeNPs.

#### 2.3.4 Mitigating cardiovascular toxicity of CRC therapy through nutritional interventions

It was found that only 4.3% of patients with newly diagnosed CRC met the American Heart Association’s ideal cardiovascular health (iCVH) criteria. Hypertension (62%), diabetes (17.2%), and overweight (BMI ≥ 25, 60.9%) were the most prevalent metabolic cardiovascular risk factors (143). Among CRC patients with comorbid cardiovascular disease and diabetes, the median survival was only 3.3 years. Cardiovascular disease was the leading non-cancer cause of death in CRC patients. Chemotherapy has well-established cardiotoxicity, and patients with pre-existing metabolic diseases are more susceptible to cardiac dysfunction (144). First-line CRC chemotherapeutic agents, such as 5-FU, leucovorin, and oxaliplatin (FOLFOX), can induce myocardial injury, hypertension, and arrhythmia. Meanwhile, overweight, high-sugar diets, and UPF may amplify oxidative stress and inflammation, significantly increasing the risk of chemotherapy-related cardiac adverse events (145).

Bioactive phytochemicals and lipids abundant in Mediterranean dietary patterns, exemplified by polyphenols and omega-3 PUFAs, exert pronounced modulatory effects on gut microbial profiles and stimulate the biosynthesis of SCFAs. SCFAs facilitate vasodilatory effects and mitigate persistent systemic inflammation through engagement with G protein-coupled receptors GPR41 and GPR43, a dual protective mechanism that collectively attenuates hypertension and the subsequent risk of cardiovascular disorders (146). A PREDIMED score of ≥9 was defined as adherence to a qualified Mediterranean diet, which was achieved by only 50.7% of patients with newly diagnosed CRC. Blood pressure and glycemic control may reduce the cardiac workload during chemotherapy. A diet rich in fruits, vegetables, and whole grains—characterized by high dietary fiber—can improve gut microbiota composition, attenuate systemic low-grade inflammation, and mitigate myocardial injury induced by chemotherapy-elicited oxidative stress. Among CRC patients adhering to the Mediterranean diet, higher rates of iCVH attainment and fewer cardiovascular events during follow-up were observed (143). Various plant polyphenols have been shown to alleviate chemotherapy-induced cardiotoxicity through their anti-inflammatory, antioxidant, and gut microbiota-modulating properties, while simultaneously inhibiting tumor progression (147).

Strict weight management can substantially reduce the risk of chemotherapy-related heart failure. Meanwhile, moderate-to-vigorous physical activity combined with a high-fiber diet may synergistically improve glucolipid metabolism and counteract the metabolic damage induced by high consumption of UPF (143). A diet rich in fiber and plant-based foods can enrich beneficial gut bacteria, thereby modulating cardiovascular oxidative stress through gut microbiota remodeling (147). Nutritional intervention can simultaneously improve risk factors for both CRC and cardiovascular disease (CVD), thereby reducing cardiovascular complications during chemotherapy as well as long-term cardiovascular mortality (148). Male CRC patients had higher rates of diabetes and overweight, necessitating a reinforced nutritional strategy emphasizing weight loss, glycemic control, and increased physical activity. Female patients achieved higher iCVH attainment rates but exhibited insufficient physical activity, warranting increased intake of dietary quality fats and dietary fiber (143).

The following clinical recommendations are proposed: (1) a modified Mediterranean diet should be prioritized throughout the entire course of chemotherapy, with strict restriction of UPF; (2) weight control should be emphasized, with targeted supplementation of anti-inflammatory plant-derived foods such as green tea; (3) blood pressure, fasting glucose, and lipid profiles should be screened at baseline and prior to each chemotherapy cycle, with concurrent adjustment of nutritional regimens; and (4) a multidisciplinary approach involving oncology, cardiology, and clinical dietetics should be implemented to jointly manage metabolic comorbidities through dietary and exercise interventions, thereby reducing the risk of chemotherapy-related cardiovascular toxicity.

### 2.4 Cardiovascular risk associated with CRC therapy: a cardio-oncology perspective

#### 2.4.1 Cardiotoxicity mechanism and clinical manifestations of chemotherapy drugs

Fluoropyrimidine agents, including 5-FU and capecitabine, constituted the backbone of CRC chemotherapy (149). However, their potential risk of severe cardiotoxic adverse effects warrants careful attention during the course of treatment. By targeting thymidylate synthase and impeding DNA synthesis, 5-FU achieved cytotoxicity against malignant cells. This pharmacological action appeared to compromise endothelial integrity, predisposing to coronary vasospastic events (150–152). Such coronary vasospasm may precipitate a spectrum of cardiac events, encompassing angina pectoris, myocardial infarction, and, in severe cases, sudden cardiac death. It has been previously shown that the incidence of 5-FU -associated cardiotoxicity ranges from approximately 3.5% (153). The period of highest risk was typically observed within 24–48 hours following the initial administration (154). As an oral prodrug of 5-FU, capecitabine appeared to share a similar mechanistic basis for cardiotoxicity with its parent compound. But, SCFAs produced by gut microbial metabolism, such as butyrate, have been demonstrated to enhance the antitumor efficacy of 5-FU. By regulating the host immune responses, short chain fatty acids can alleviate chemotherapy related adverse reactions (155, 156). These findings provided a novel theoretical basis for mitigating the cardiotoxicity of chemotherapeutic drugs in CRC therapy.

Oxaliplatin-based combination chemotherapy regimens were commonly employed in clinical practice for the treatment of CRC. Its cardiac toxicity was mainly manifested as arrhythmia, especially QT interval prolongation. Oxaliplatin appeared to disrupt the electrophysiological stability of cardiomyocytes through oxidative stress and mitochondrial injury, thereby predisposing to arrhythmogenic events (157). The combination of oxaliplatin and 5-FU appeared to produce a synergistic exacerbation of cardiotoxicity. This might be associated with the superimposition of distinct myocardial injury mechanisms induced by the two drugs (158, 159). Current evidence indicated that the composition of the gut microbiota can modulate both the metabolism and toxicity profiles of chemotherapeutic agents. The abundance of specific intestinal bacteria, for instance Akkermansia muciniphila, has been positively correlated with chemotherapy responsiveness. It may affect drug efficacy and toxicity by regulating host immunity and metabolic pathways (160). In addition, gut microbiota metabolites such as butyrate not only enhanced the anti-tumor effect of 5-FU (155), but may also protected myocardial cells by regulating mitochondrial autophagy (161). This mitigated oxaliplatin-induced cardiac damage. In clinical practice, close electrocardiographic monitoring, particularly of QT interval changes, should be performed in CRC patients receiving oxaliplatin-based regimens. On the other hand, modulation of the gut microbiota may offer an alternative strategy to optimize therapeutic outcomes in CRC patients.

Anti-angiogenic agents such as bevacizumab exerted crucial effects in the treatment of CRC by inhibiting the VEGF signaling pathway. Bevacizumab has been associated with hypertension (reported in up to 30% of treated patients), thromboembolic events, and heart failure (162, 163). This toxicity profile was attributable to impaired vasodilation and endothelial dysfunction consequent to VEGF inhibition (164, 165). For CRC patients requiring prolonged bevacizumab exposure, vigilant monitoring of blood pressure and cardiac function was essential. Notably, gut microbiota dysbiosis is closely associated with the onset and progression of hypertension and cardiovascular diseases (166). Patients with CRC were characterized by significant alterations in the diversity and composition of gut microbiota. Pathogen expansion (*E. coli, enterotoxigenic B. fragilis*) coupled with loss of butyrate-producing commensals may drive systemic inflammatory amplification. The vascular toxicity and myocardial injury associated with antiangiogenic agents were amplified (167). Furthermore, SCFAs produced by gut microbiota metabolism, particularly butyrate, exerted anti-inflammatory and blood pressure-regulating effects (168). Butyrate deficiency may perturb vascular homeostasis, undermining blood pressure stability (169–171). Microbiota-targeted dietary strategies during VEGF inhibition may offer a novel cardioprotective adjunct to antiangiogenic regimens. For instance, supplementation with probiotics such as *Bifidobacterium* species has been demonstrated to ameliorate chemotherapy-induced toxicities through modulation of intestinal immunity and production of anti-inflammatory mediators (172).

#### 2.4.2 Long term damage and dose-response relationship of radiotherapy to the heart

Pelvic radiotherapy constitutes a standard therapeutic modality for locally advanced rectal cancer. During the radiotherapy process, the heart is inevitably exposed to scattered radiation, which may lead to a series of long-term cardiac toxicity events such as coronary microvascular damage, myocardial fibrosis, and pericarditis (173). Such pathological alterations serve as key biological drivers behind the 30% higher long-term cardiovascular disease risk detected in patients 10 to 15 years following pelvic radiotherapy (174). Accordingly, as clinical teams aim to attain satisfactory local tumor control in rectal cancer patients, longitudinal comprehensive assessment and surveillance of pelvic radiotherapy-related cardiotoxic risks should be routinely implemented throughout long-term follow-up.

Specifically, there exists a distinct dose-response correlation between pelvic radiation exposure and subsequent cardiac injury in rectal cancer patients. Clinical cohort data indicates that every 1 Gy increment in mean cardiac radiation dose is associated with a 7.4% elevated risk of severe cardiovascular adverse events, including myocardial infarction and congestive heart failure (175). Furthermore, patients’ pre-existing cardiovascular and metabolic comorbidities (hypertension and type 2 diabetes included) exert interactive effects with pelvic radiation exposure, and these two factors collectively modulate long-term clinical prognosis (176).

#### 2.4.3 Targeted therapy and immunotherapy related cardiovascular adverse events

Anti-epidermal growth factor (EGFR) monoclonal antibodies including cetuximab exert anti-tumor effects by inhibiting EGFR-mediated proliferative signaling, yet they possess off-target toxic effects that trigger direct cardiomyocyte damage. A meta-analysis of patients diagnosed with RAS wild-type metastatic CRC revealed that cetuximab-combined chemotherapy correlates with substantially higher risks of cutaneous toxicities and renal adverse reactions, with hypomagnesemia being the most prevalent renal electrolyte disturbance (177). As compared with cetuximab, panitumumab, a second anti-EGFR monoclonal antibody, confers greater odds of grade ≥3 severe hypomagnesemia, revealing significant inter-agent disparities in electrolyte toxicity profiles (178). Accordingly, routine surveillance of serum magnesium concentrations and immediate intervention to reverse hypomagnesemia throughout anti-EGFR therapeutic administration are mandatory to reduce the risk of life-threatening arrhythmias.

ICIs including pembrolizumab elicit potent anti-tumor responses through the stimulation of cytotoxic T lymphocytes, whereas these agents carry the risk of ICI-associated myocarditis, a life-threatening immune-mediated cardiovascular adverse event. A published case report documented a patient with CRC who developed multiple severe adverse events including myocarditis following administration of an anti-PD-1 inhibitor (sintilimab) combined with regorafenib (179). Another patient diagnosed with locally advanced colon carcinoma who received trastuzumab monotherapy developed successive immune-related adverse events (irAEs), manifesting sequentially as myasthenia gravis, myocarditis and rhabdomyolysis (180). These clinical cases highlight the severe nature of ICI-related myocarditis and indicate that clinicians should maintain a high degree of vigilance.

### 2.5 Gut microbiota and cardio-oncology

#### 2.5.1 Gut microbiota metabolites as direct regulators of the cardiovascular system

The metabolites produced by the gut microbiota of CRC patients played a critical and complex role in modulating host cardiovascular homeostasis. First, trimethylamine N-oxide (TMAO), generated by gut microbial metabolism of dietary choline and L-carnitine, has been conclusively linked to increased risks of atherosclerosis, thrombosis, and cardiovascular disease (181). TMAO accelerated the progression of atherosclerosis by promoting platelet activation and foam cell formation (182, 183). Chemotherapy-associated gut dysbiosis in CRC patients may elevate TMAO levels abnormally, thereby increasing cardiotoxicity risk. This suggested that TMAO may represent a significant biomarker connecting CRC treatment to cardiovascular complications. Secondly, SCFAs, with butyrate being the most prominent, played a cardioprotective role in preserving cardiovascular homeostasis. Butyrate and related SCFAs lowered blood pressure and prevented myocardial hypertrophy by engaging G protein-coupled receptors GPR41 and GPR43 (184, 185). However, patients with CRC frequently underwent substantial intestinal dysbiosis. This was primarily manifested by a reduced abundance of butyrate-producing bacteria (e.g., Roseburia intestinalis), leading to diminished butyrate synthesis (186). This alteration in microbial community structure attenuates the cardioprotective effects of SCFAs, potentially exacerbating cardiovascular risk in CRC patients (187, 188). Research indicated that microbiota dysbiosis induced by high-fat feeding further attenuates the cardioprotective functions of propionate and related SCFAs. It could potentiate lymphatic metastasis of CRC via suppression of the GPR41 signaling cascade. This indirectly attested to the indispensable role of SCFAs in sustaining intestinal and cardiovascular homeostasis (17).

Bile acid metabolites served as dual modulators of myocardial energy homeostasis and contractile function. The intestinal microbiota mediated the deconjugation and metabolic transformation of bile acids via bile salt hydrolase (BSH) expression, generating secondary bile acids including deoxycholic acid (189). These metabolites finely regulated cardiac energy metabolism and contractile function through ligand-mediated activation of the farnesoid X receptor (FXR) and G protein-coupled bile acid receptor 1 (TGR5) (190, 191). Patients with CRC frequently exhibited gut microbiota dysbiosis that disrupted bile acid metabolic homeostasis, generating abnormal secondary bile acid profiles that subsequently exert deleterious effects on cardiac function (192). For example, patients after cholecystectomy often developed dysbiosis and metabolic disorders from altered bile acid enterohepatic circulation, increasing their cardiovascular disease risk (193). Moreover, the crosstalk between gut microbiota and cholesterol metabolism represented a crucial nexus in CRC and cardiovascular disease. Dysbiotic alterations could trigger a deleterious feedback loop by modulating bile acid and oxysterol metabolic networks, thus propelled disease progression (22). Thus, targeted modulation of microbial metabolites, encompassing TMAO, SCFAs, and bile acids, was expected to constitute a promising new strategy for concurrently ameliorating cancer prognosis and cardiovascular function in CRC patients.

#### 2.5.2 The gut microbiota-driven inflammatory-immune axis in cardiac injury

Pro-inflammatory microbial taxa represented by Fusobacterium nucleatum and toxin-producing *Bacteroides fragilis* facilitate the progression of CRC via sustained induction of chronic inflammatory responses and elevated oxidative stress (70, 194, 195). Chronic persistent systemic inflammatory cascades constitute a key pathogenic driver of myocardial damage. Gut microbial metabolites represented by secondary bile acids and TMAO are well-documented risk factors for cardiovascular complications, wherein aberrant cholesterol homeostasis serves as a shared pathogenic mechanism bridging CRC and cardiovascular disease (22).

Intestinal microbiota dysbiosis in patients with CRC constituted a pivotal characteristic underlying tumorigenesis and neoplastic progression. Such microbial imbalance was not only restricted to local gut pathology but also could propagate to distal organs, notably the heart, through the induction of systemic inflammatory reactions. The characteristic of intestinal microbiota imbalance was the abundance of pathogenic bacteria such as *Clostridium difficile*. These pathogenic bacteria impaired the integrity of the intestinal epithelial barrier (196). The impairment of this barrier function resulted in increased intestinal permeability, enabling the translocation of bacterial products, such as endotoxins, into the systemic circulation (3). Circulatory endotoxin exemplified by LPS, served as the classical ligand that activates the Toll-like receptor 4 (TLR4) signaling cascade. Activation of the TLR4 signaling pathway triggered downstream pro-inflammatory cascades, including NF-κB, thereby induced cardiomyocyte apoptosis and promoted myocardial interstitial fibrosis (197). This pathological process was regarded as a critical mechanism contributing to the aggravation of chemotherapy-associated cardiac toxicity. Microorganisms including *Fusobacterium nucleatum* were capable of regulating the immunological microenvironment through the TLR4/NF-κB signaling axis (198). Cardiomyocytes within this milieu become more vulnerable to chemotherapy-induced injury, thereby amplifying cardiotoxicity (199). Disruption of the ‘gut permeability’–endotoxin–TLR4 signaling axis represented a hallmark of intestinal ecological dysregulation in patients with CRC. This was also one of the pathophysiological mechanisms underlying myocardial injury induced by colorectal neoplasms. The critical role of the intestinal microbiota in host immunity was manifested in its modulation of the equilibrium between T helper 17 (Th17) cells and Treg cells (200, 201). Among CRC patients, this homeostatic balance was commonly perturbed. Microbial dysbiosis can drive excessive activation and expansion of pro-inflammatory Th17 cells, concomitant with a relative reduction in immunosuppressive Treg cells (202–204). In models of CRC hepatic metastasis, *Fusobacterium nucleatum* colonization altered the hepatic immune landscape, manifested as decreased Th17 lymphocyte infiltration alongside elevated Treg cell aggregation (205). When such T cell imbalance occurred in the heart, diminished Treg-mediated immunosuppression compromised cardiac immune tolerance, whereas augmented Th17 activity and interleukin-17 (IL-17) secretion may drive myocardial tissue infiltration (206). IL-17 served as a powerful pro-inflammatory mediator that mobilized and activated neutrophils and allied immune cells. And then, exacerbation of cardiac inflammation promoted progressive myocardial fibrosis and adverse structural remodeling. Thus, the perturbation of Th17/Treg homeostasis elicited by intestinal dysbiosis in CRC constitutes another critical immunological axis that intensified myocardial injury and interstitial fibrosis by fostering sustained IL-17-mediated inflammatory responses.

Among the enteric pathogenic species associated with CRC pathogenesis, *Fusobacterium nucleatum* assumed a particularly conspicuous role. Its tumor-promoting mechanisms were intimately linked to inflammasome priming and activation, processes that may confer cardiotoxicity. The NOD-like receptor protein 3 (NLRP3) inflammasome in host cells could be activated by *Fusobacterium nucleatum (*207). The assembly of the NLRP3 inflammasome mediated the cleavage and activation of Caspase-1, thereby promoting the maturation and released of potent pro-inflammatory cytokines, including interleukin-1β (IL-1β) and interleukin-18 (IL-18) (208). In CRC, chronic elevation of IL-1β and IL-18 levels across the tumor microenvironment and circulatory system propagated and amplified systemic inflammatory responses. The gut microbiota-driven inflammatory response, operating through the NLRP3–IL-1β/IL-18 axis, chronically impacted cardiac function and structure. It instigated and worsened myocardial inflammation, cellular degeneration, and progressive ventricular remodeling. Notably, enrichment of *Fusobacterium nucleatum* in CRC tissues has been associated with unfavorable patient prognosis (209). Individuals with cancer who were subjected to radiation and chemotherapy already face a significantly increased susceptibility to the development of heart failure (210). Thus, the heightened post-treatment heart failure risk may be mechanistically linked to *Fusobacterium nucleatum*-driven NLRP3 inflammasome activation, thereby amplified inflammatory signaling. This contributed a microbiota-centered inflammatory mechanism to the conceptual framework of cardio-oncology.

#### 2.5.3 The regulatory potential of nutritional intervention on the gut heart axis

SCFAs derived from the microbial fermentation of dietary fiber exert dual protective effects: they diminish the risk of CRC via enhanced fecal bulk and optimized glycemic homeostasis, while concurrently attenuating dyslipidemia and chronic systemic inflammation to alleviate subsequent cardiovascular disease susceptibility (211, 212). Dysbiosis of gut metabolic microbiota, marked by elevated TMAO generation and compromised biosynthesis of SCFAs, accelerates the development of both CRC and atherosclerotic lesions, establishing a common pathogenic pathway underlying the co-occurrence of colorectal malignancy and cardiovascular complications (213, 214). For intervention against the aforementioned pathological cascade, selected probiotic strains represented by specific *Lactobacillus* isolates suppress microbial trimethylamine biosynthesis within the gut lumen to decrease peripheral TMAO accumulation, which further relieves the cumulative pathological burden of cardiovascular complications (215). Population-based epidemiological investigations demonstrate that adequate dietary consumption of probiotic supplements, prebiotic fibers, and fermented yogurt correlates with a 50% relative decrease in the incident risk of CRC (216). Furthermore, β-glucan, a prebiotic polysaccharide abundant in dietary fibers, fosters the expansion of favorable intestinal commensals and restrains the excessive reproduction of enteric pathogens. This balanced gut microecology further mediates potent immunomodulatory and anti-inflammatory responses, ultimately diminishing the susceptibility to CRC and cardiovascular complications (217).

During CRC treatment, nutritional intervention conferred cardioprotective effects through the modulation of gut microbiota and its metabolites. Diets rich in polyphenols, such as those abundant in berries and green tea, have been demonstrated to reduce TMAO levels by promoting the proliferation of beneficial bacteria, including *Akkermansia* and *Lactobacillus (*218). As an atherogenic mediator synthesized by intestinal microbiota from dietary choline and L-carnitine, TMAO exhibited elevated circulating levels that were strongly correlated with increased incidence of cardiovascular adverse events (219). Through TMAO inhibition, polyphenolic substances diminished systemic inflammation and, leveraging their direct antioxidant and anti-inflammatory bioactivities, protected myocardial cells from oxidative injury and cardiotoxicity induced by chemotherapy drugs, including irinotecan (220). Moreover, polyphenols can fortify intestinal epithelial barrier integrity, curtailed endotoxin leakage into the bloodstream, and subsequently alleviated cardiac inflammatory reactions (221, 222).

Omega-3 PUFAs, exemplified by EPA and DHA, possessed important functionality in gut microbiota modulation and myocardial protection. The ingestion of omega-3 PUFAs significantly enriches butyrate-producing bacterial populations, specifically *Faecalibacterium prausnitzii* and *Roseburia*, resulted in increased butyrate generation in the gut (115). Butyric acid, served as a critical short-chain fatty acid, exhibited robust anti-inflammatory and anti-fibrotic bioactivities. It attenuated myocardial fibrosis by activating the peroxisome proliferator-activated receptor gamma (PPAR-γ) signaling axis, which in turn suppressed fibroblast activation and prevented excessive collagen deposition within the myocardial interstitium (223, 224). Omega-3 fatty acids offered a potential nutritional intervention strategy for mitigating chemotherapy-related cardiotoxicity through the “microbiota–butyrate–PPAR-γ” axis.

Accumulating evidence has delineated the correlations between gut microbiota and nutrition, gut microbiota and CRC, as well as nutrition and cardiovascular toxicity induced by CRC therapies. Nevertheless, clinical investigations focusing on the three-way interaction among nutrition, gut microbiota, and cardiovascular events in patients with CRC remain scarce. Future research is warranted to further dissect the interactive regulatory network of these three factors.

### 2.6 Targeting gut microbiota improved CRC prognosis and cardiovascular outcomes

#### 2.6.1 Prospects for fecal microbiota transplantation in CRC therapy

In patients with CRC, FMT was employed for intestinal microbiota reconstruction. Transplantation of healthy donor fecal microbiota directly reversed chemotherapy- or radiotherapy-induced intestinal dysbiosis (225, 226). By enriching *Roseburia* intestinalis and driving butyrate production, FMT augmented radiation-triggered autophagic tumor cell death and sensitized tumors to radiotherapy via OR51E1/RALB axis activation (227). Through gut microbiota modulation, *Akkermansia muciniphila* enrichment, and intestinal barrier restoration, FMT inhibited CRC progression (202). Beyond direct tumor growth suppression, FMT potentiated existing treatments through immune microenvironment remodeling. FMT-mediated gut microbiota restoration decreased systemic TMAO and endotoxemia, offering protection against chemotherapy-related cardiotoxicity (228). Gut microbiota converted choline and L-carnitine into TMAO. By rebalancing TMAO-producing microbiota (e.g., specific *Clostridium* spp.), FMT may lower cardiovascular disease risk. However, rigorous donor screening was a prerequisite for FMT. Pathogenic or opportunistic donor bacteria (e.g., *pks+E. coli, enterotoxigenic B. fragilis*) transmitted through FMT increased recipient infection and tumor recurrence risk (2, 229). Hence, exhaustive pathogen screening of donors was a prerequisite for FMT to guarantee procedural safety.

Most current FMT studies were small-scale or preclinical, with no long-term data on tumor recurrence or cardiovascular events within years post-treatment (230, 231). Baseline characterization and metabolomic analysis of the recipient gut microbiome may inform FMT strategies (195). In sum, FMT is a valuable CRC strategy: it modulates microbiota, enhances efficacy, and reduces cardiac toxicity. Clinical translation still faces safety, standardization, and long-term efficacy challenges.

#### 2.6.2 Mimics of microbial metabolites and cardioprotective drug development

Dietary fiber was fermented by gut microbiota to produce butyrate and other SCFAs, which were essential for intestinal homeostasis and anti-tumor immunity (51, 58). The anti-inflammatory and anti-fibrotic effects of butyrate were mediated through GPCR activation (GPR43/GPR109A) and HDAC inhibition (232, 233). Butyrate induced CRC cell apoptosis and enhanced chemosensitivity (234). CRC-associated Porphyromonas-derived butyrate may promote tumorigenesis by inducing cellular senescence (235). Therefore, the development of butyrate analogues, such as phenylbutyrate, aims to mimic the beneficial effects of butyrate (e.g., GPR43 activation, HDAC inhibition) while circumventing its potential pro-tumorigenic risks. These analogues reduced chemotherapy-induced heart damage via anti-inflammatory and antifibrotic effects, and enhanced antitumor efficacy through immune microenvironment regulation.

### 2.7 Future directions and translational challenges

Multi-omics analysis can be employed to track functional microbiome dynamics throughout the therapeutic stages of CRC. Integrative analysis of serum metabolomics and gut microbiome metagenomics revealed that the combination of Firmicutes and threonic acid in the intestinal flora could distinguish CRC patients from healthy controls (236). Applying such multi-omics integration strategies to longitudinal monitoring before and after CRC treatment holds promise for identifying microbiota-metabolite drivers closely associated with the temporal onset of cardiotoxicity (e.g., acute myocardial injury, chronic heart failure). A key challenge is modeling causality between microbial community dynamics and cardiotoxicity onset using longitudinal cohorts. Mendelian randomization studies have established causal relationships between specific bacterial genera (e.g., *Anaerotruncus*, *Intestinibacter*) and CRC (237). These models not only facilitate the identification of patients most likely to derive clinical benefit, but also enable dynamic adjustment of intervention regimens, thereby achieving true precision medicine.

Single-cell sequencing technology unlocks unparalleled resolution to decode the molecular mechanisms whereby microbial metabolites inflict cardiac damage on specific cell subtypes. Dysbiosis-altered metabolites in diabetic cardiomyopathy cardiotoxicity may drive post-myocardial injury inflammation and fibrosis by modulating cardiac macrophage polarization (M1 vs. M2) and T cell differentiation (238). The integrative application of single-cell RNA sequencing and spatial transcriptomics enables the localization of specific cell subpopulations and their signaling pathways regulated by microbiota-derived metabolites at the single-cell level. This provided precise molecular targets for the development of microbiota-heart axis-targeted intervention strategies (e.g., specific probiotics, FMT) (46).

## 3 Limitation

This review has several limitations. First, the systematic literature search was restricted to PubMed/MEDLINE, with additional studies identified via manual screening of reference lists. Accordingly, relevant articles indexed solely in other bibliographic databases may have been omitted. Second, most supporting evidence is derived from observational studies and preclinical animal models, while high-quality clinical trials and causal inference analyses regarding the gut microbiota–nutrition–heart axis in CRC patients remain insufficient. Third, significant heterogeneity exists across included studies in terms of tumor subtypes, therapeutic regimens, nutritional intervention protocols, and cardiac toxicity assessment criteria, restricting standardized quantitative synthesis. Fourth, current research mainly focuses on single-factor linear correlations, lacking systematic multi-omics analyses to unravel complex interactive regulatory networks among gut microbial metabolites, inflammatory pathways and cardiovascular toxicity. Finally, microbiota-targeted interventions such as FMT and probiotics lack large-scale long-term clinical validation, and confounding cardiometabolic comorbidities in enrolled patients are not uniformly adjusted in primary studies, which may compromise the robustness of the conclusions.

## 4 Conclusion

CRC is not merely a focal malignancy; its development constitutes a systemic process tightly coupled with intestinal dysbiosis, metabolic abnormalities, and cardiovascular toxicity. TMAO and SCFAs served as molecular executors of the gut microbiota’s dual pathogenic role in tumor progression and cardiac injury. Nutritional intervention reshaped microbial community structure while exerting dual antitumor and cardioprotective effects. This marks a paradigm shift in CRC management from single-target therapy to multi-system collaborative regulation.

Emerging therapeutic strategies, including FMT and precision probiotics, have demonstrated promising potential. Yet their efficacy and safety require validation through high-quality randomized controlled trials. Future research should integrate multi-omics data, elucidate causal mechanisms, and overcome bench-to-bedside barriers, thereby improving both the quality of life and long-term prognosis of CRC patients.
